# Fruit and Vegetable Consumption among Community Dwelling Elderly in an Iranian Population

**Published:** 2010

**Authors:** Ali M. Sabzghabaee, Parisa Mirmoghtadaee, Mehdi Mohammadi

**Affiliations:** 1 Isfahan Clinical Toxicology Research Center, Isfahan University of Medical Sciences (IUMS), Isfahan, Iran; 2 Department of Clinical Pharmacy, IUMS, Isfahan, Iran; 3 School of Pharmacy & Pharmaceutical Science, IUMS, Isfahan, Iran

**Keywords:** Fruit consumption, Vegetable, Elderly

## Abstract

**Objectives:**

Fruits and vegetables are important components of a healthy diet, and their consumption could help prevent a wide range of diseases. In this study, fruit and vegetable consumption in elderly people were assessed.

**Methods:**

This cross-sectional study was conducted among elderly (≥65 year-old) people who came to pharmacies affiliated with Isfahan University of Medical Science. Face-to-face interview using a questionnaire including food frequency questionnaire and demographic and socioeconomic variables was administered.

**Results:**

Of the total 504 participants, 56.3% were male and 43.7% were female. The mean daily serving of fruit and vegetable (combined) consumption in men and women were 4.58±1.31 and 4.65±1.28, respectively. The prevalence of daily fruit and vegetable intake of 5 or more servings was 37.9%. Low educational and low income participants ate lower fruits and vegetables (combined). Age, gender, smoking, and chronic disease had no significant influence on their consumption. Educational level was the only independent predictor of fruit and vegetable consumption (OR: 3.81, CI: 1.64-8.84).

**Conclusions:**

Most elderly people consumed less than the recommended levels of fruits and vegetables. From the point of view of prevention of chronic disease, health education programs which targeted elderly people particularly for those at the risk of low consumption are needed and recommended.

## INTRODUCTION

High consumption of fruits and vegetables reduces many chronic diseases such as stroke, cardiovascular disease, metabolic disease and some cancers.^1^–^7^ They contain essential vitamins, minerals, fibers and other bioactive compounds.^8^ The World Health Organization (WHO) recommends the consumption of at least 400 g, or at least 5 servings of fruits and vegetables a day.^9^ Increasing percentage of a population who eat enough fruits and vegetables is the healthy people 2010 objective in US and other guidelines.^9^–^11^ To improve fruit and vegetable intake in a community, health policy makers should identify the correlates of fruit and vegetable consumption especially for at risk population to design proper interventional programs. Socioeconomic circumstances, physical factors and psychological wellbeing are factors which affect fruit and vegetable consumption.^2^,^12^ With increasing in the elderly population, successful or healthy aging has been paid enough attention in most societies.^13^ Iran is one of the developing countries at risk of aging.^14^ In aging people, therapeutic and preventive actions of fibers against constipation and hemorrhoids, diarrhea, colon cancer, breast cancer and other malignancies, vascular diseases and hypertension, and gallstone formation seem to be important.^1^ All national dietary guidelines underline the necessity to increase dietary fiber intake, and therefore fruits and vegetables for the elderly population. To help health policy makers to design interventions to increase fruit and vegetable consumption in a population, it is necessary to determine not only the current level of intake in the elderly people but also its associated factors. In the current study, we determined the prevalence of fruit and vegetable consumption and some socioeconomic correlates in individuals aged 65 year-old and elder in Isfahan, Iran.

## METHODS

This cross-sectional study was conducted among 504 randomly selected elderly people (65 year-old and more) who came to pharmacies affiliated with Isfahan University of Medical Science in Isfahan, Iran in 2007-2008 in autumn, winter and spring. It was approved by Research Committee of Isfahan University of Medical Science. Verbal assent was obtained from participants. The elders who entered 3 educational pharmacies to take their or related families’ prescriptions or non-prescriptions were included in the study. The participants who could not speak due to language or disease were excluded from the study. Face-to-face interviews were conducted with the whole sample using a structured questionnaire on some demographic and socioeconomic variables such as sex, age, smoking, educational level, monthly income, chronic disease and amount and type of fruit and vegetable consumption in a day. Smokers were defined as occasional smokers and current smokers.^15^ Presence of chronic diseases was according to participants answers and WHO diseases classification.^16^ Ten nutrition specialists validated this questionnaire (both face and content validity). In the present study, we represented the groups of vegetables and fruits with 12 and15 items, respectively. They were the most common fruits and vegetables in our community which were available. The responses were based on standardized servings for each item.^17^ To calculate how many elderly individuals ate fruit and vegetable, the responses of each individual that indicated the daily intake of each item were selected, corresponding to the following frequency categories: one to two, three to four and five or more times a day. The sum of each item was calculated and thus, the number of fruits, vegetables, fruits and vegetables (combined) per individual were obtained and finally, categorized as daily fruit and vegetable (combined) intake of less than 5 servings and 5 or more servings. According to some guidelines, consumption of 2 or more servings of fruits and 3 or more servings of vegetables were considered as standard.^9^,^10^

### Statistical Analysis

SPSS for Windows software (version 17, SPSS Chicago, IL) was used for statistical analysis. Descriptive data were expressed as means ± standard deviation. After assessment of normal distribution by KolmogorovSmirnov test, chisquare test was used to assess fruit and vegetable consumption according to some demographic and socioeconomic variables. Logistic regression analysis was conducted with fruit and vegetable consumption as the dependent variable and educational level (lower or higher than high school) and monthly income (lower or higher than 435$) as predictors variables.

## RESULTS

Characteristics of the participants are presented in Table 1. Mean age of our sample was 71.92 ± 5.94 years. The prevalence of fruit and vegetable consumption 5 or more servings/day was 38.7% in men and 36.8% in women. Eating 2 or lower servings/day was observed in 3.2% of the elders. The mean daily serving of fruit and vegetable (combined) was 4.58 ± 1.31 and 4.65 ± 1.28 in men and women, respectively. The mean daily fruit and vegetable consumption separately was 1.86 ± 0.68 and 2.74 ± 0.83, respectively. The elders who were 74 year-old or more ate less fruits and vegetables than 65-74 year-old but the difference was not significant. Fruit consumption, vegetable consumption, fruit and vegetable consumption (combined) according to ages, genders, income and educational levels are shown in Table 2. Fruits, vegetables, fruit and vegetable consumption (combined) were statistically different according to educational levels (P<0.01). The difference of fruit, fruit and vegetable (combined) consumption was significant in low and high monthly income groups (P<0.01 and P=0.04, respectively). Vegetable intake was not different according to the monthly income. There were no associations between fruit and vegetable consumption and smoking status, gender, age, and chronic disease. After regression analysis, educational level remained the independent predictor of higher consumption of fruit and vegetable (OR: 3.81, 95% CI: 1.64-8.84).

**Table 1:** Characteristics of the study population

Variables	% of participants
**Gender**	
Male	56.3
Female	43.7
**Age (years)**	
65-74	69.4
75 and older	30.6
**Smoking**	
Yes	11.9
No	88.1
Monthly income*	
Lower than the poverty red line	86.1
Higher than the poverty red line	13.9
**Education** (years)	
Less than 12	92.7
More than 12	7.3
**Chronic disease**	
Yes	93.1
No	6.9
**Fruit and vegetable consumption** (servings/day)	
4 or less	62.1
5 or more	37.9
**Fruit consumption** (servings/day) (Mean ± SD)	
1 or less	55.8
2 and more	44.2
**Vegetable consumption** (servings/day)	
2 or less	62.1
3 and more	38.9

* According to the unofficial report of provincial authorities, poverty red line was considered as monthly income of less than 435 US $ for an average family with4 members living in Isfahan city.

**Table 2:** Fruit and vegetable consumption (isolated and combined) in different groups

		Fruit consumption (Serving/day) (Mean ± SD	P value*	Vegetable consumption (Serving/day) (Mean ± SD)	P value	Fruit and vegetable consumption (Serving/day) (Mean ± SD)	P value
Age (years)	65 -74	1.87±0.85	0.45	2.76±0.86	0.56	4.64±1.30	0.44
	75 and more	1.82±0.74		271±0.74		4.54±1.28	
Sex	Men	1.84±0.66	0.48	2.73±0.83	0.7	4.58±1.31	0.54
	Women	1.88 ± 0.7		2.76±0.83		4.65 ± 1.28	
Smoking	Yes	1.89±0.59	0.72	2.71±0.59	0.76	4.60±0.92	0.98
	No	1.85±.69		2.75±0.85		4.61±1.31	
Educational evel	Lower than high school	1.82 ± 0.82	0.02	2.70 ± 0.79	0.001	4.52 ± 1.27	0.000
	Higher than high school	2.14 ± 0.54		3.06 ± 0.63		5.23 ± 1.33	
Monthly income	Lower than poverty red line	1.83 ± 0.66	0.01	2.72 ± 0.82	0.12	4.55 ± 1.29	0.01
	Higher than poverty red line	2.04 ± 0.75		2.89 ± 0.86		4.94 ± 1.26	

* P value <0.05 was considered significant

## DISCUSSION

To the best of our knowledge, this was the first study of its kind in Iran. Of special interest in the context of this study was the high prevalence of consumption of 4 or less servings of fruits and vegetables per day in this population while one third of the participants achieved WHO recommendations regarding daily servings of fruits and vegetables. This provides alarming evidence for policy makers and health care professionals to pay more attention to improve this concern. Although the fruit and vegetable consumption by elderly population in Iran is higher than that in some developing and developed countries but it is far from nutritional objectives in the health guidelines.^9^,^10^,^18^ In a Brazilian study in 2005, it was found that among the individuals aged 65 years or older, only 20.6% of the women and 14.8% of the men consumed five or more servings of fruits and vegetables per day.18 In the United States, the overall prevalence of consumption of fruits or vegetables or both five or more times per day was 24.7% among adults. In 1998, it was 37% and 51% among urban and rural old respondents, respectively.10 In the present study, inadequate fruits and vegetables intake was strongly associated with low level of education in elders. It is consistent with several studies in other countries and different aged groups.^10^,^18^–^21^ Education which could be effective on income/ socioeconomic status has been shown to have one of the strongest influences on fruit and vegetable intake in the United States and France among adults.^20^ Low level of education can affect intake due to the adoption of inadequate dietary habits. In addition, low socioeconomic groups generally have a more restrictive food budget, and prefer more energydense and satisfying foods. But, in line with demographic and socioeconomic variables, chewing and swallowing problems, diseases such as depression and dementia and motion difficulties are some reasons of low consumption of fruits and vegetables, which should be considered in designing programs to improve nutrition among elderly people.^19^ As in many developing countries, Iran is facing rapid nutritional transition. Taking high caloric and low fiber foods has been prevalent in our community since the last decade and threatens all aged groups.^22^,^23^ Then, fruit and vegetable consumption will decrease more and more in future. Clearly, there is a vital need to establish a national strategy for integrating preventive measures including lifestyle modification, notably dietary changes including higher consumption of fruits and vegetables and lower taking of fatty foods. The main limitation of this study was its crosssectional nature that might reduce the strength of clinical significance of correlations. In addition, because this analysis reports consumption according to the number of times per day that fruits and vegetables are eaten, the results may over or underestimate the calculated proportion. To determine more accurate estimation of fruit and vegetable consumption in this community, a study in healthy elders with no chronic disease and measurement of serum biomarkers related to vegetables and fruits are recommended. High prevalence of chronic disease might reduce fruit and vegetable consumption in our studied population.
